# Supplementary material for: Overcoming Ligand Discovery Challenges: Developing Peptide-Based Tracers for SPSB2
Source: ACS Chem Biol. 2025 Dec 8;21(2):274–83. doi: 10.1021/acschembio.5c00702 (PMC12930380; doi:10.1021/acschembio.5c00702)
Supplement: Supplementary file 1 [file cb5c00702_si_001.pdf]

Supporting Information

Overcoming Ligand Discovery Challenges: Developing Peptide-Based Tracers for SPSB2

Christopher Lenz[1,2], Lewis Elson[1,2], Johannes Dopfer[1,2], Frederic Farges[1,2], Andreas Krämer[1,2], Frank Löhr[3], Susanne Müller[1,2], Stéphanie M. Guéret[4], Herbert Waldmann[5,6], Volker Dötsch[3], Krishna Saxena[1,2]*, Stefan Knapp[1,2]*

[1]Institute for Pharmaceutical Chemistry, Johann Wolfgang Goethe-University, Max-von-Laue-Straße 9, 60438 Frankfurt am Main, Germany

[2]Structural Genomics Consortium, Buchmann Institute for Life Sciences (BMLS), Max-von-Laue-Straße 15, Johann Wolfgang Goethe-University, 60438 Frankfurt am Main, Germany

[3]Institute of Biophysical Chemistry and Center for Biomolecular Magnetic Resonance, Goethe-University, Max-von-Laue Str. 9, 60438 Frankfurt, Germany

[4]Medicinal Chemistry, Research and Early Development, Cardiovascular, Renal and Metabolism, Biopharmaceutical R&D, AstraZeneca, 43183 Gothenburg, Sweden

[5]Department of Chemical Biology, Max-Planck-Institute of Molecular Physiology, 44227 Dortmund, Germany

[6]Faculty of Chemistry and Chemical Biology, TU Dortmund University, 44227 Dortmund, Germany

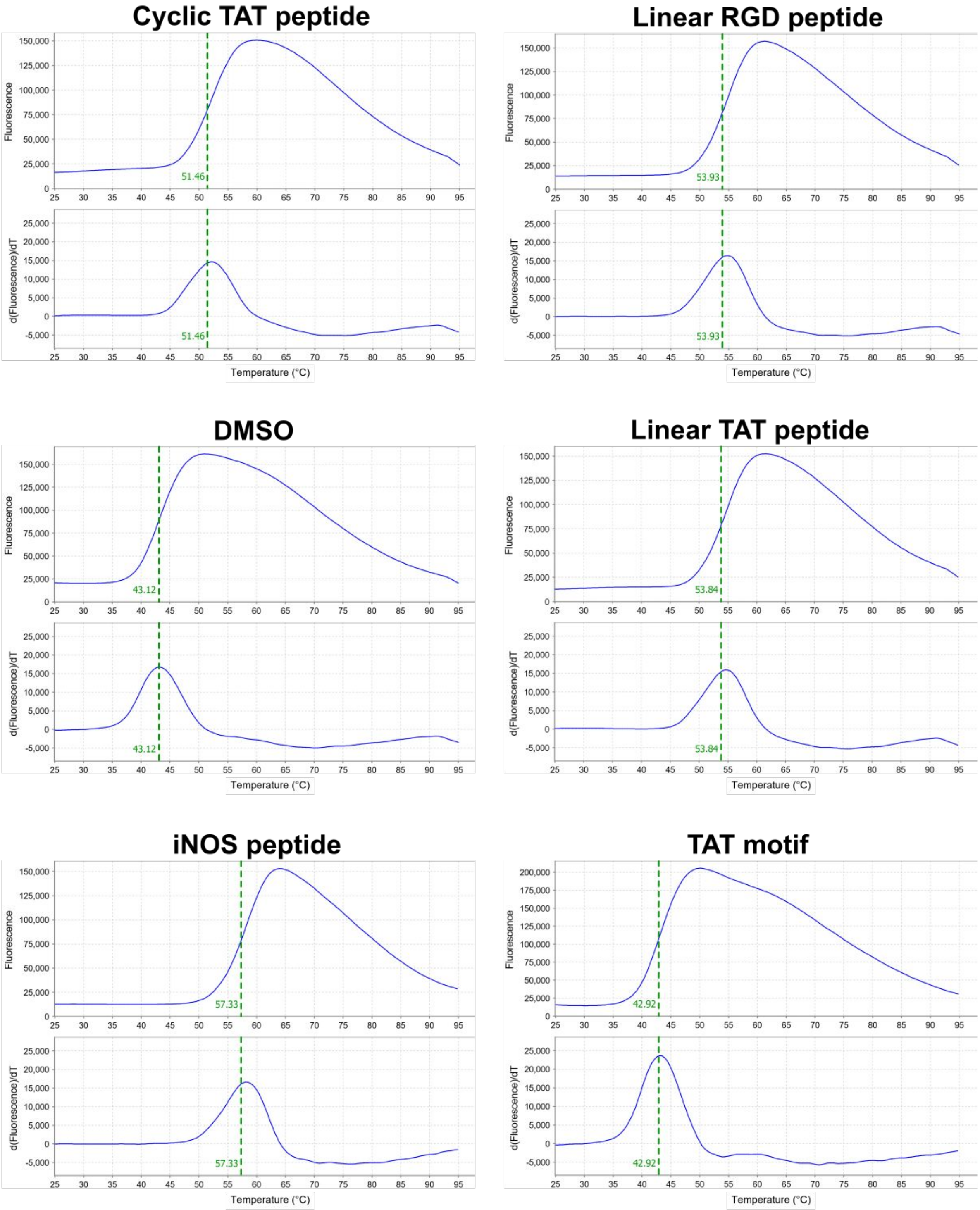

Figure S1. Exemplary thermal shift melting curves (top panels) and their corresponding derivative curves (bottom panels) for $SPSB2^{SPRY}$ with different peptides or the DMSO control. The Boltzmann-fit T_m is indicated by a green dotted line.

Supplement Table 1. Overview of SPR single cycle kinetics results. K_D, k_{on} and k_{off} values are depicted as mean ± SD (n = 3). N.D: Values were not determined due to weak or apparent two-phase binding.

**Peptide**	**SPR K_D [nM]**	**SPR k_{on} [$M^{-1}s^{-1}$]**	**SPR k_{off} [s^{-1}]**
Linear RGD peptide	162.0 ± 4.0	$(2.0 \pm 0.1)*10^{5}$	$(3.2 \pm 0.1)*10^{-2}$
iNOS peptide	0.8 ± 0.1	$(2.9 \pm 0.1)*10^{6}$	$(2.4 \pm 0.1)*10^{-3}$
Linear TAT peptide	N.D.	N.D.	N.D.
Cyclic TAT peptide	N.D.	N.D.	N.D.
TAT motif	N.D.	N.D.	N.D.

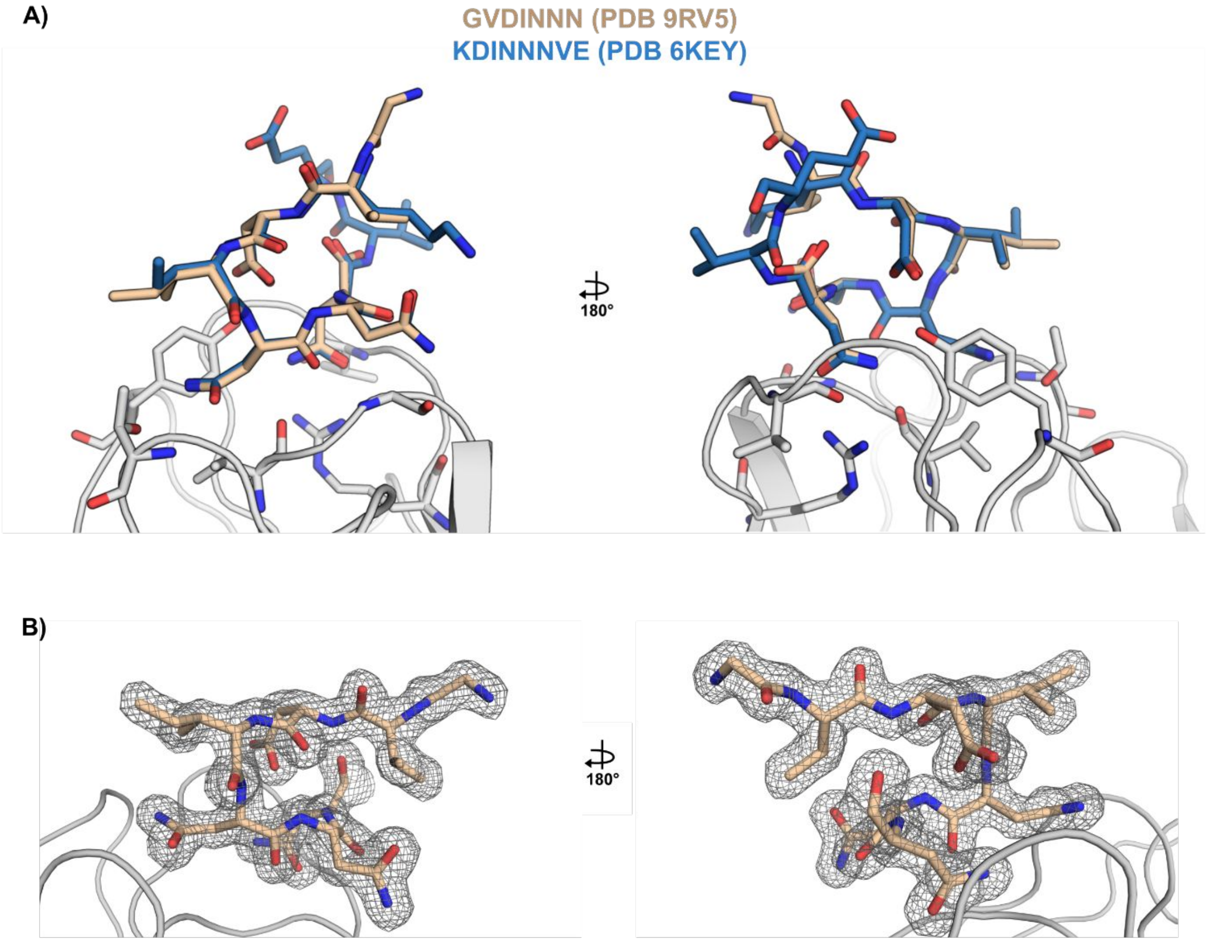

Figure S2. A) Superimposition of crystallized linear TAT peptide sequence GVDINNN in complex with hSPSB2^{SPRY} (beige, PDB ID: 9RV5) and KDINNNVE (blue, PDB ID: 6KEY). B) Electron density of PDB: 9RV5 and modelled peptide in complex with SPSB2^{SPRY}.

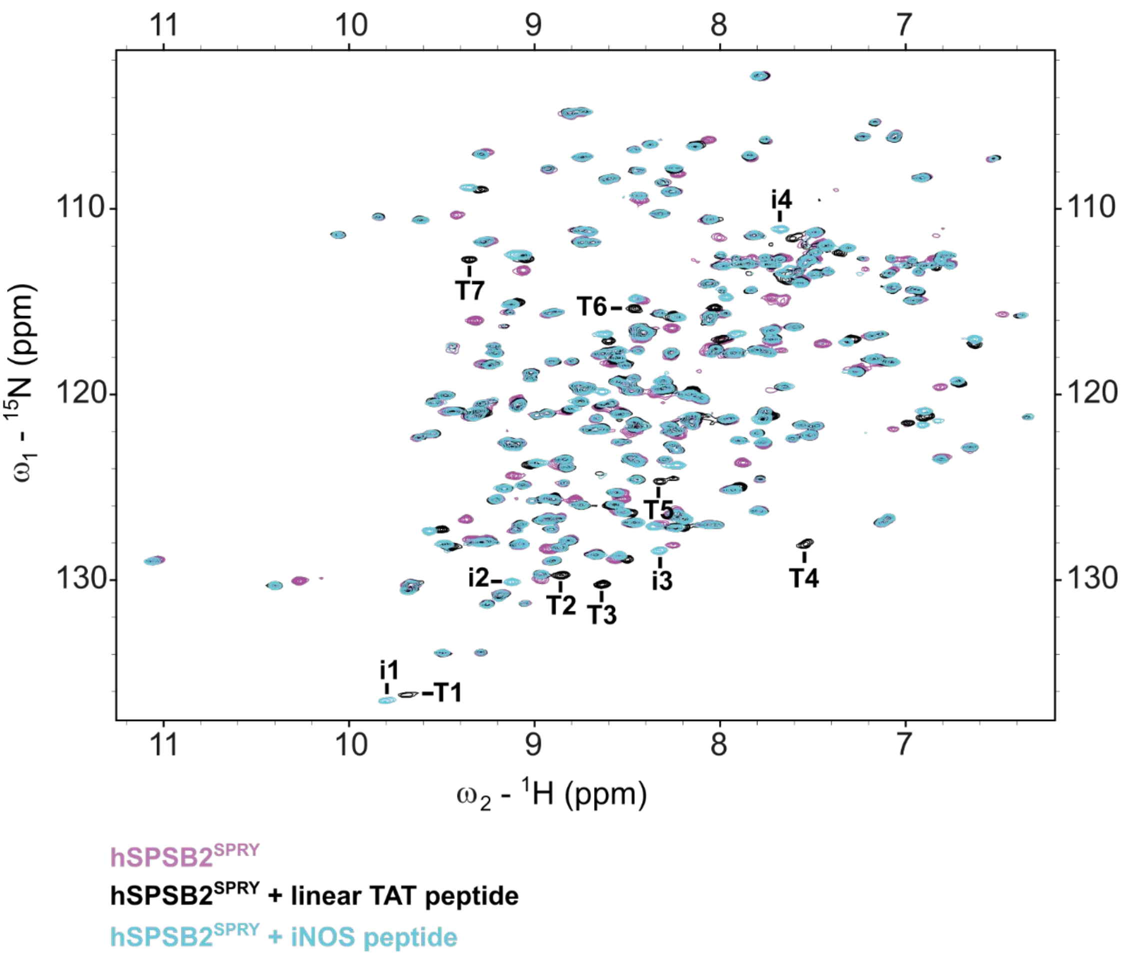

Figure S3. ^{1}H-^{15}N BEST-TROSY spectra of ^{15}N labelled hSPSB2^{SPRY} in the absence (magenta) and presence of linear TAT peptide (black) or iNOS peptide (cyan). Exemplary peaks that exhibit distinct chemical shift perturbations compared to the apo form and do not overlap are labelled as **i#** for the iNOS peptide and **T#** for the linear TAT peptide.

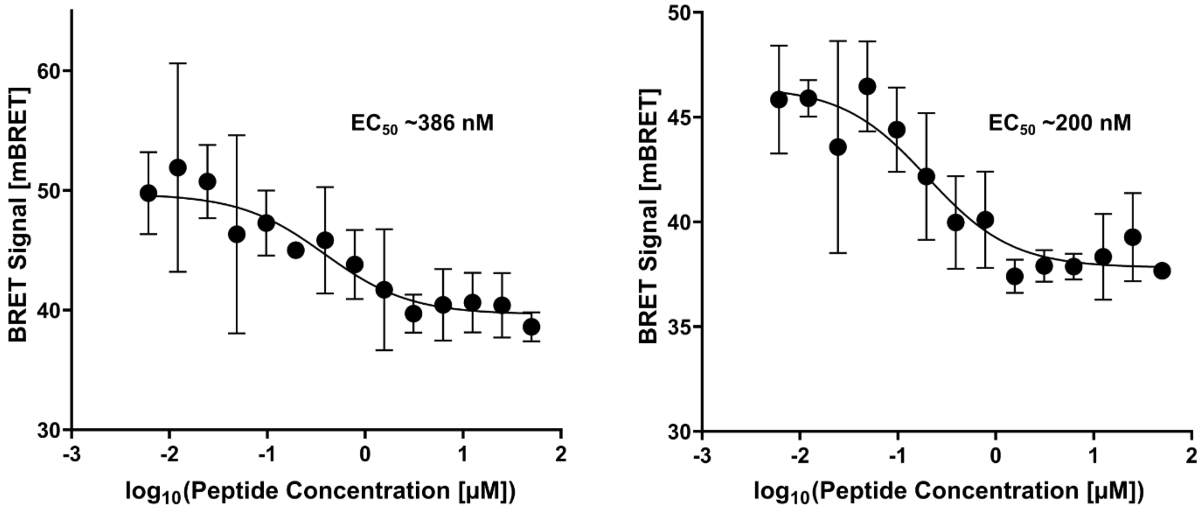

Figure S4. Displacement NanoBRET data of 30 nM cyclic TAT tracer (left) and 20 nM cyclic TAT tracer (right) with cyclic TAT peptide in intact HCT116 cells with respective EC_{50} values. Measured values are depicted as mean ± SD (n = 3).

Supplement Table 2. Data collection and refinement table

**Data collection**	**SPSB2 SPRY - linear TAT peptide**
Beamline	DLS I03
Wavelength (Å)	0.97625
Space group	$P\ 1\ 2_1\ 1$
Cell dimensions	
a, b, c (Å)	40.47, 90.11, 57.82
α, β, γ (°)	90.00, 97.47, 90.00
Resolution (Å)*	48.37-1.75 (1.78-1.75)
unique observations*	41203 (2193)
R_{meas}*	0.144 (0.901)
Completeness (%)*	99.7 (97.7)
Multiplicity*	7.0 (6.3)
mean I/σI*	8.7 (1.9)
Wilson B-factor ($Å^2$)	15.1
$CC_{1/2}$*	0.997 (0.799)
**Refinement**	
R_{work} / R_{free}	17.13 / 19.89
Atoms (all, chain, peptide solvent)	3405, 2999, 104, 302
Average B-factors all atoms ($Å^2$)	20.0
Rms deviations	
Bond lengths (Å)	0.006
Bond angles (°)	1.431
Ramachandran (%)	
Favoured / Outlier	98.0 / 0.0
**Protein Data Bank entry**	**9RV5**

*Values for the highest resolution shell are shown in parentheses.
